# Efficient and organic host–guest room-temperature phosphorescence: tunable triplet–singlet crossing and theoretical calculations for molecular packing†

**DOI:** 10.1039/d1sc01175h

**Published:** 2021-04-05

**Authors:** Yunxiang Lei, Junfang Yang, Wenbo Dai, Yisha Lan, Jianhui Yang, Xiaoyan Zheng, Jianbing Shi, Bin Tong, Zhengxu Cai, Yuping Dong

**Affiliations:** School of Materials Science & Engineering, Beijing Institute of Technology Beijing 100081 P. R. China caizx@bit.edu.cn; School of Chemistry and Materials Engineering, Wenzhou University Wenzhou 325035 P. R. China; Department School of Chemistry and Chemical Engineering, Beijing Institute of Technology Beijing 100081 P. R. China xiaoyanzheng@bit.edu.cn

## Abstract

Organic host–guest doped materials exhibiting the room temperature phosphorescence (RTP) phenomenon have attracted considerable attention. However, it is still challenging to investigate their corresponding luminescence mechanism, because for host–guest systems, it is very difficult to obtain single crystals compared to single-component or co-crystal component materials. Herein, we developed a series of organic doped materials with triphenylamine (TPA) as the host and TPA derivatives with different electron-donating groups as guests. The doped materials showed strong fluorescence, thermally activated delayed fluorescence (*τ*: 39–47 ms), and efficient room temperature phosphorescence (*Φ*_phos_: 7.3–9.1%; *τ*: 170–262 ms). The intensity ratio between the delayed fluorescence and phosphorescence was tuned by the guest species and concentration. Molecular dynamics simulations were used to simulate the molecular conformation of guest molecules in the host matrix and the interaction between the host and guest molecules. Therefore, the photophysical properties were calculated using the QM/MM model. This work provides a new concept for the study of molecular packing of guest molecules in the host matrix.

## Abstract 2

Molecular dynamics simulations were used to simulate the molecular conformation and interaction between hosts and guests. This work provides a new concept for the study of molecular packing for the investigation of the luminescence mechanism.

## Graphical abstract

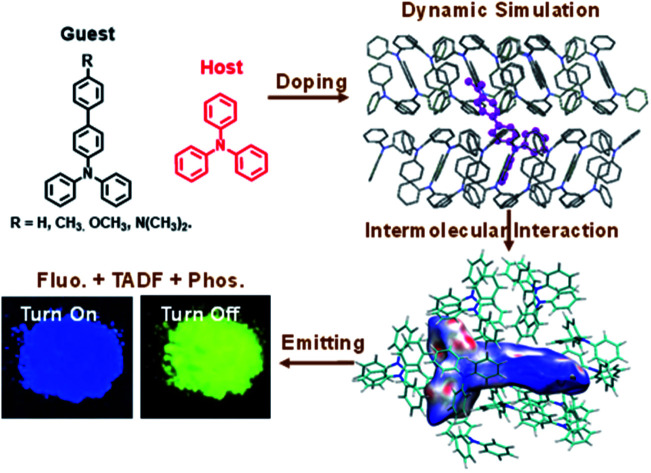

## Introduction

Organic room temperature phosphorescent (RTP) materials with long afterglow emission have attracted much attention owing to their unique advantages including structural diversity, multiple functionality, and biocompatibility.^1–8^ RTP materials with long lifetimes exhibited a wide range of applications in the fields of anti-counterfeiting, data security, and biological imaging.^9–12^ Recently, the luminescence mechanism of single-component phosphorescent materials has been investigated. In general, the RTP properties of pure organic molecules are highly dependent on intermolecular interactions in the solid state. Strong intermolecular interactions can overcome the spin-forbidden transitions between the excited singlet and triplet states to realize an efficient intersystem crossing (ISC) process and can stabilize excited triplet states, promoting the persistent RTP effect.^13–15^ Various building blocks including carbazole, dibenzothiophene, and 9,9-dimethylxanthene have been successfully utilized as core units in the construction of single-component RTP luminogens.^16–24^ The inherent mechanism and the relationship between the molecular structure and packing performance can be easily investigated, since their crystal structures are easily obtained and molecular packing can be tuned *via* molecular design.

Compared with single-component RTP materials, the host–guest doped system or co-crystal system can convert two non-phosphorescent components into excellent RTP materials.^25–31^ The ease of the preparation process facilitates the ever expansive library of RTP materials and provides insights into the host–guest system. However, exploration of host–guest RTP systems for application as organic opto-electronic materials is still in its infancy due to the lack of a clear understanding of the luminescence mechanism. Wang *et al.* showed that the host matrix of the doped system only restricts the motion of guest molecules, thus decreasing the non-radiative decay and promoting radiation transition of triplet excitons.^20^ Adachi *et al.* found that the rigid host matrix can act as an oxygen barrier to avoid triplet quenching. Recent studies also demonstrated that the host matrix plays a role in energy synergy in phosphorescence emission.^25–29^ However, because of trace amounts of guest molecules (<1%) in the doped system, traditional characterization methods, such as single crystal X-ray diffraction, scanning electron microscopy (SEM), and transmission electron microscopy (TEM), are considered unsuitable for accurate investigation of the specific molecular conformation of the guest molecules in the host matrix. As a result, researchers have been unable to perform exact theoretical calculations of the corresponding photophysical properties of the guest molecules in the doped systems, limiting further development of host–guest RTP materials.

**Fig. 1:**
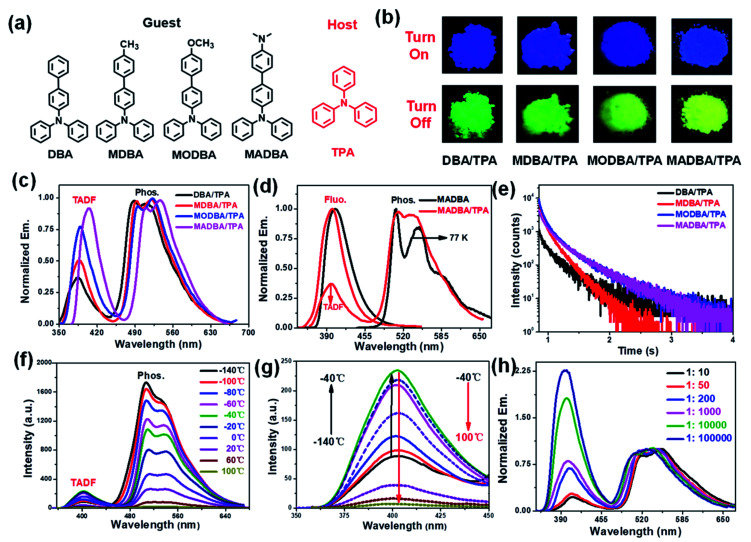
(a) The molecular structures of the guest and host molecules. (b) The fluorescence (top) and phosphorescence (down) images of the four host–guest doped materials (excitation wavelength: 360 nm). (c) Delayed emission spectra of host–guest doped materials (delay time: 5 ms). (d) Prompt and delayed spectra of the MADBA guest and MADBA/TPA doped materials (MADBA: solution state, 1.0 × 10^−4^ mol L^−1^, Ex.: 360 nm, 77 K). (e) Delayed decay curves of the host–guest doped materials at an emission wavelength of 520 nm (Ex.: 360 nm). (f) Delayed emission intensity of the MADBA/TPA material at different temperatures (Ex.: 360 nm, delay time: 5 ms). (g) TADF intensity of the MADBA/TPA material at different temperatures. (h) Delayed emission spectra of the MADBA/TPA materials with different amounts of MADBA (molar ratio, Ex.: 360 nm, delay time: 5 ms).

In this work, we provide an approach to investigate molecular packing in host–guest systems. A series of organic RTP materials with triphenylamine (TPA) bearing perfect symmetry and high crystallinity as the host and four triphenylamine derivatives (DBA, MDBA, MODBA and MADBA) with different electron-donating groups as the guests (Fig. 1a, Scheme S1†) are developed. The molecular structures of the host and guest compounds are similar and both have electron donating properties. The close energy levels between the host and guest molecules facilitate energy transfer. Therefore, all doped materials exhibited three different emissive performances, namely, fluorescence, thermally activated delayed fluorescence (TADF), and phosphorescence. The total luminescence efficiency is up to 80%. It is worth noting that the species and concentration of the guests can adjust ISC and reverse intersystem crossing (RISC) processes, thereby tuning the relative intensity of TADF and RTP. Importantly, since the molecular configuration of the guest molecules in the host matrix cannot be measured using current technical methods, we first simulated the molecular conformations of the guest molecules in the host matrix using molecular dynamics (MD) simulations and then comprehensively investigated the intermolecular interactions between the host and guest molecules. Furthermore, the photophysical properties of the guest molecules in different environments were calculated using the hybrid quantum mechanics/molecular mechanics (QM/MM) method. The obtained results strongly imply the possibility of realizing efficient and robust RTP from pure organic host–guest systems, thus paving the way for future studies of the molecular conformations and spatial intermolecular interactions of doped guest molecules in the host matrix.

## Results and discussion

According to our previous work, the host matrix is believed to play a synergistic role in energy transfer during the phosphorescence emission of guest molecules, and the smaller energy gaps between the excited state energy levels of the host and guest molecules are obviously beneficial for energy transfer.^32–35^ Therefore, four TPA derivatives (DBA, MDBA, MODBA, and MADBA) with electron donating groups were designed and synthesized as the guests (Fig. 1a, ESI Scheme S1†). All prepared guests have strong electron donating properties and similar molecular structures, which are conducive to similar excited state energy levels between each other. Similar absorption and fluorescence curves are observed for DBA, MDBA, and MODBA. However, the maximum absorption and fluorescence wavelengths of DBA, MDBA, and MODBA are significantly shorter than those of MADBA (ESI Fig. S1a†). All guests exhibit strong blue fluorescence (389–404 nm) in solution (Table 1, ESI Fig. S1c†), but green phosphorescence with emission peaks of 585–502 nm can only be detected at 77 K (ESI Fig. S1d†). The melting point of TPA is 124 °C, hence, the doped materials were prepared by the melt-casting method in a nitrogen atmosphere (the molar ratio of guest to host is 1 : 1000).

**Table 1:** Photophysical properties of the guests and doped materials^a^

Sample	Fluo.			Phos.		
	*λ*_em_ (nm)	*Φ*_F_ (%)	*τ* (ns)	*λ*_em_ (nm)	*Φ*_P_ (%)	*τ* (ms)
DBA^b^	387	43	1.8	486^c^, 520^c^	13.2^c^	545^c^
MDBA^b^	392	45	1.9	488^c^, 523^c^	10.7^c^	623^c^
MODBA^b^	395	47	2.2	492^c^, 525^c^	12.4^c^	661^c^
MADBA^b^	404	51	2.3	508^c^, 542^c^	11.8^c^	728^c^
DBA/TPA	385	76	2.4	386^d^, 517	7.3	45^d^, 170
MDBA/TPA	389	77	1.8	388^d^, 521	8.1	39^d^, 181
MODBA/TPA	392	75	2.1	390^d^, 525	8.6	40^d^, 192
MADBA/TPA	403	70	2.3	405^d^, 538	9.2	42^d^, 262

a Guest : host = 1 : 1000 (molar ratio).

b In THF solution, 1.0 × 10^−4^ mol L^−1^.

c At 77 K.

d Delayed fluorescence; excitation wavelength: 360 nm for phosphorescence and 320 nm for fluorescence.

TPA crystalline powder only exhibited weak fluorescence at 380 nm (ESI Fig. S3a†). Upon doping with 0.1% guest molecules, it exhibited strong blue fluorescence with the maximum peaks between 385 nm and 403 nm (Fig. 1b, ESI Fig. S3b†), which correspond to those of the guests (Table 1). The fluorescence quantum yields (*Φ*_fluo._) of the four doped materials are all greater than 70%, which are much higher than the fluorescence intensities of the guests in tetrahydrofuran (THF) solvent (Table 1). This result demonstrates that the TPA matrix can greatly restrict the non-radiative decay process of the guest molecules. Since both the host and guest show similar energy levels, intermolecular charge transfer cannot occur, which in turn results in no new absorption peaks (ESI Fig. S4†). More importantly, the four host–guest materials display a visible yellow-green afterglow once the irradiation source is removed, indicating the existence of RTP properties (Fig. 1b). As shown in Fig. 1c, all four doped materials exhibited three peaks in their delayed time spectra around 390 nm, 500 nm, and 520 nm, respectively. The high energy bands (385–403 nm) can be attributed to delayed fluorescence, because they are comparable to that of the corresponding guest molecules (Fig. 1c, Table 1). The two delayed emission peaks at 500 nm and 520 nm are attributed to the phosphorescence of the guest molecules, resulting from the guest phosphorescence peaks at low temperatures (Fig. 1d, Table 1). As shown in Fig. 1e and Table 1, all materials show long phosphorescence lifetimes ranging from 0.17–0.26 s and high phosphorescence efficiency with quantum yields between 7% and 9% (Table 1), which increase with the increasing donating ability of the guest molecules. To further investigate the delayed fluorescence, temperature variation experiments of delayed fluorescence spectra were carried out (Fig. 1f). As the temperature increases from −140 °C to 100 °C, the phosphorescence intensity of MADBA/TPA greatly decreases, which is consistent with the previous results.^24,32^ However, the intensity of the delayed fluorescence at 400 nm wavelength first increased (from −140 °C to −40 °C), indicating the existence of the RISC of excitons,^36–38^ and then decreased with increasing temperature from −40 °C to 100 °C due to elevated thermal intramolecular vibrational motions (Fig. 1f).^37,38^ Moreover, the TADF lifetimes of the four doped materials can reach up to 39–45 ms (ESI Fig. S5,†Table 1), which exceed those of most delayed fluorescence materials.^36–38^ Therefore, excitons have very efficient ISC and RISC capabilities.

**Fig. 2:**
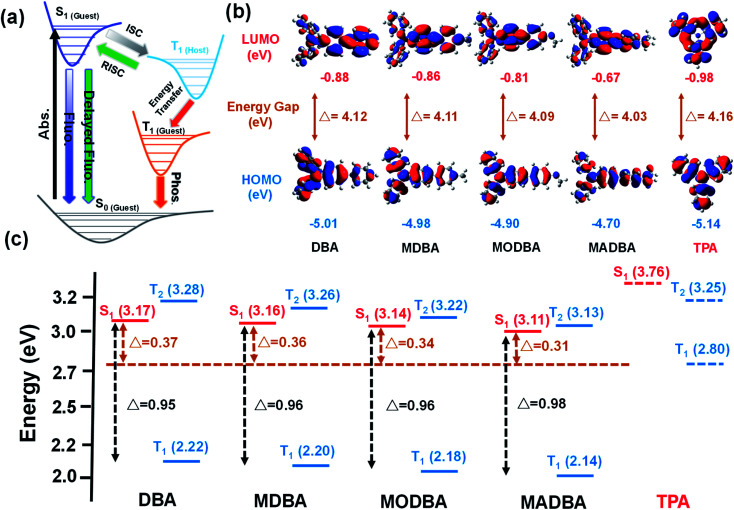
(a) Proposed transfer path between the guest and host. (b) The LUMO and HOMO of guest and host molecules. (c) The energy levels of the guests and host.

Interestingly, when the guest changes from DBA to MADBA, the TADF intensity of the doped material gradually increases (Fig. 1c), indicating that ISC and RISC processes can be tuned *via* variations of the guest molecules. To further investigate ISC and RISC processes in doped systems, quantum calculations were carried out using density functional theory (DFT) (Fig. 2). The phosphorescence emission in a host–guest system is primarily determined by the energy levels of the host and guest molecules (Fig. 2a).^32–35^ As shown in Fig. 2b, the highest occupied molecular orbitals (HOMOs) and the lowest unoccupied molecular orbitals (LUMOs) of all guest molecules are distributed on the entire molecule. The energy gaps decrease gradually with increasing electron-donating ability of the groups connected to the TPA moiety. In addition, the results are in good agreement with the absorption wavelengths of all guest molecules (ESI Fig. S1†). No phosphorescence was observed in pure guest powder, because the large energy gaps between the S_1_ and T_1_ of guest molecules do not facilitate ISC. However, the T_1_ of the host can act as a bridge between the S_1_ and T_1_ of the guest molecules (Fig. 2c).^32–35^ Moreover, from DBA to MADBA, the energy gaps between the S_1_ of the guest molecule and the T_1_ of the host TPA molecule gradually decrease. The reduced energy gaps are beneficial for ISC and RISC of excitons, increasing the TADF and RTP intensity of the doped materials (Fig. 1c, Table 1). Therefore, MADBA/TPA exhibits optimal TADF and RTP performance. Furthermore, since the concentration of the guest molecules plays an important role in the RTP properties of doped materials,^24,30–34^ a series of MADBA/TPA doped materials with different molar ratios were prepared. As the ratio of MADBA increases, the phosphorescence emission of the host–guest materials exhibits a slight red shift and the intensity ratio between TADF and RTP gradually decreases (Fig. 1h). This is probably due to the molecular aggregation caused by the high concentration of the guest molecules. Notably, at MADBA concentration as low as 0.0001 mol% (1 : 100 000), the host–guest doped materials still exhibited an obvious visible afterglow, indicating the high efficiency of our host–guest luminescent systems.^24,31–35^

**Fig. 3:**
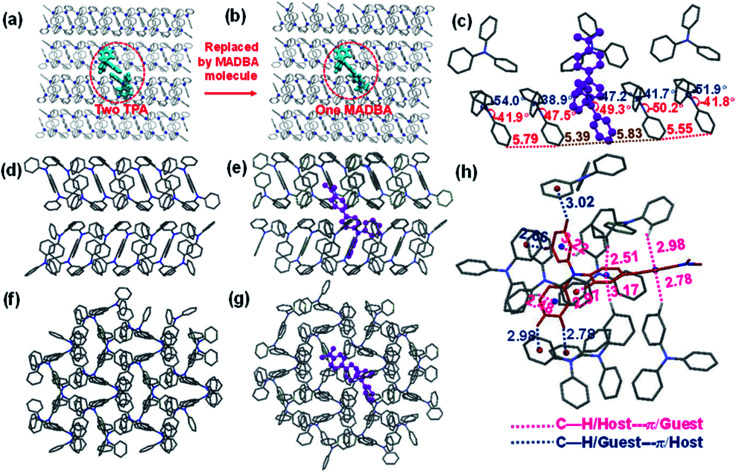
(a and b) Model setup of the MADBA/TPA doped system. (c) The molecular configuration of the simulated MADBA/TPA doped system. (d) Molecular packing along the *b*-axis in the TPA single crystal. (e) Molecular packing along the *b*-axis of the simulated MADBA/TPA doped system. (f) Molecular packing along the *c*-axis of the TPA single crystal. (g) Molecular packing along the *c*-axis of the simulated MADBA/TPA doped system. (h) The interaction distance of C–H⋯π between a MADBA molecule and surrounding host molecules. The distances between each phenyl ring center of the MADBA molecule and the hydrogen atoms of the TPA molecules are labelled with a blue line. The corresponding distance between the phenyl ring center of TPA and the hydrogen atoms of MADBA are labelled with a pink line.

**Fig. 4:**
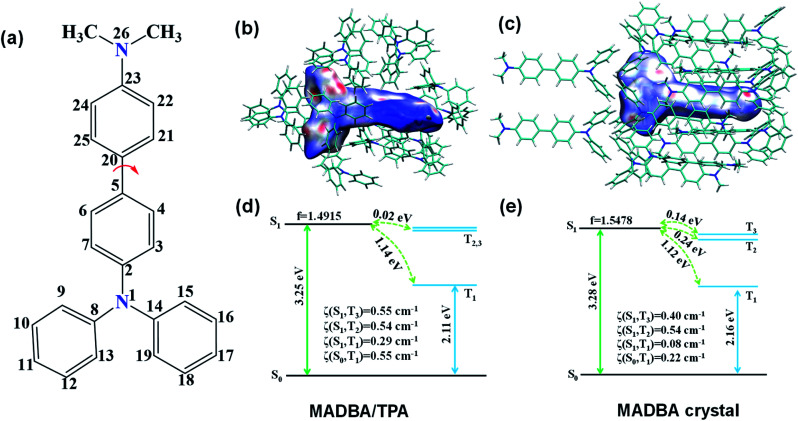
(a) The number of carbon atoms in the MADBA guest molecule. (b) Hirshfeld surface for the MADBA/TPA system mapped with a dnorm distance. The C–H⋯π interactions are shown in red on the surface. (c) Hirshfeld surface for the MADBA crystal state mapped with a dnorm distance. The C–H⋯π interactions are shown in red on the surface. (d and e) The calculated energy level diagram, spin–orbit couplings (*ξ*) between singlet and triplet states, and the oscillator strengths of the S_1_ state in the crystal based on the optimized first triplet-state geometries using the ONIOM method for the doped system and MADBA crystalline state.

The analysis of the single crystal structure of materials by using an X-ray diffractometer is currently the available method to observe organic compounds at the molecular level. The high molar ratio of guest to host molecules in doped materials prevents obtainment of single crystals that contain both host and guest molecules. Therefore, although researchers have developed a variety of doped systems, the specific configuration and molecular packing of the guest molecules in the host matrix are still unknown. In order to address the inherent issues in this research field, further theoretical calculations were performed to simulate the doped configurations and spatial molecular packing of the guest molecule in the host matrix using MD simulations. MADBA/TPA with the best RTP performance was selected. The initial MADBA/TPA model was set up based on the TPA crystal. As shown in Fig. 3a and b, two TPA molecules are removed from the TPA crystal and the vacancy is filled with a single MADBA molecule, which is larger in size than the TPA molecule. The obtained MADBA/TPA model possesses a 1 : 190 molar ratio of MADBA to TPA. Starting from the initial MADBA/TPA configuration, we performed further 10 ns production MD simulations to relax the whole system using the GROMACS software package (version 5.1.5, details in the ESI†).^39^ Compared with the conformations of the host molecule TPA in a single crystal, the corresponding conformations of the host molecules adjacent to the MADBA molecule doped in the MADBA/TPA system are different, and the twist angles show an obvious increase after doping (marked in red, Fig. 3c and S8†). This is due to the guest molecule MADBA with a larger spatial volume being compressed into the spatial position of the original host molecules. However, even though there is a very small proportion of MADBA guests in the doped system, it has minimal impact on the overall arrangement of the host matrix, and the stacking of TPA molecules of the simulated doped system is almost comparable to that of the single crystal (Fig. 3d–g). XRD results also confirmed that the arrangement of the TPA host before and after doping with guest molecules is almost unchanged (Fig. S9†). Therefore, it is reasonable and feasible to simulate the microstructure of the doped system by MD simulations. According to the simulated MADBA/TPA model, we analyzed the relative spatial positions of the MADBA molecule and its surroundings, as well as intermolecular interactions (Fig. 3h, S10,†Fig. 4b–e). As shown in Fig. 3h, the space between the MADBA guest molecule and its surroundings is relatively dense. Importantly, multiple C–H⋯π interactions between the host and guest molecules are observed with short distances between them (2.487–3.319 Å), and the average distance between MADBA and surrounding host molecules is only 2.86 Å. Particularly, the distances between the TPA moiety of MADBA and the surrounding host molecule TPA are relatively close, which implies stronger interactions in the doped system. Therefore, the non-radiative decay channel after doping with MADBA can be effectively restricted. To further investigate the intrinsic mechanism of the MADBA/TPA system, we set up a cluster model based on the above simulated MADBA/TPA model and performed QM/MM calculations to study the photophysical properties using the Gaussian 16 package^40^ (ESI Fig. S7†). In order to better reproduce the experimental results, five functionals, namely, B3LYP,^41^ BMK,^42^ CAM-B3LYP,^43^ M06-2X^44^ and ωB97XD,^45^ were chosen to perform benchmark tests and select the most appropriate functional to deal with the current system. The B3LYP functional shows the optimal performance. Here the doped MADBA molecule was selected as the QM region and calculated at the B3LYP/6-31G** level, whereas the other TPA molecules were treated as the MM region by the universal force field.^46^ During QM/MM geometry optimization, the QM region was fixed and the MM region was allowed to move. The results show that after doping the MADBA molecule into the TPA host matrix, the structural changes of MADBA from S_0_ to T_1_ in the doped state are obviously smaller than those in the solution state, as shown in Fig. S9, Tables S1 and S2.† Therefore, the reorganization energy in the doped system is also smaller than that in solution. The dihedral angles of MADBA in the doped system become smaller, such as C4–C5–C20–C21 decreases from 31.47° in the solution state to 16.90° in the doped state. To further understand the influence of the surrounding host molecules on the MADBA molecule, distances between MADBA and its surroundings in the doped system were compared (Fig. 3h), as well as the corresponding distances of pure single crystal MADBA (Fig. S11†), and the average distance between MADBA and surrounding MADBA molecules is only 3.49 Å. Therefore, the doped system has much closer distances (2.86 Å) than those of the pure MADBA crystal, reflecting stronger interactions between host and guest molecules in the doped system. In order to visualize the intermolecular interaction strength, Hirshfeld surface calculations^47,48^ were performed using the Multiwfn program.^49,50^ As shown in Fig. 4b and c, the Hirshfeld surface exhibits the evolution of intermolecular interactions in the MADBA/TPA system and MADBA crystal. Decomposition of the Hirshfeld surface reveals that the intermolecular C–H⋯π interactions of the doped system are greater than those of the pure MADBA crystal, owing to the large area distribution of the red region on the Hirshfeld surface of the central MADBA molecule. In addition, based on the optimized electronic structure of the triplet state, the energy gaps and spin–orbit couplings (SOCs) between S_1_ and low-lying triplet states also support the easier ISC process of the doped system than that in the pure MADBA crystal. This is due to the smaller energy gaps and larger SOCs in the doped system than in the MADBA crystal, see Fig. 4d and e. For example, the SOC between S_0_ and T_1_ of the doped system is 0.55 cm^−1^, and the corresponding value of the MADBA crystal is only 0.22 cm^−1^. The corresponding SOC between S_1_ and T_1_ for the doped system, 0.29 cm^−1^, reduces to only 0.08 cm^−1^ in the MADBA system. Therefore, doping trace amounts of MADBA is beneficial to promote ISC of excitons, thereby leading to phosphorescence emission in the host matrix.

## Conclusions

In this work, a series of organic doped materials with TPA as the host and TPA derivatives with different electron-donating groups as the guests were developed. The doped materials showed strong fluorescence, TADF, and RTP emission. The intensity ratio between TADF and RTP could be adjusted by either the guest species or concentration. Moreover, molecular dynamics simulations were employed to simulate the molecular conformation and spatial location of guest molecules in the host matrix. To the best of our knowledge, this is the first use of molecular dynamics simulations to study doped materials. The photophysical properties of the guest molecules were calculated using the QM/MM model. This work provides a new concept for studying the specific molecular packing of guest molecules in the host matrix by molecular dynamics simulations.

## Author contributions

Y. D., Z. C. and X. Z. designed the research work and revised the manuscript. Y. L. synthesized the materials and wrote the manuscript. J. Y. carried out density functional theory calculations and wrote the manuscript. Y. L., W. D., J. Y. and Y. L. carried out photophysical property measurements. J. S. and B. T. edited the manuscript. All authors discussed the results and commented on the manuscript.

## Conflicts of interest

There are no conflicts to declare.

## Supplementary Material

SC-012-D1SC01175H-s001
