# Supplementary material for: Efficient and organic host–guest room-temperature phosphorescence: tunable triplet–singlet crossing and theoretical calculations for molecular packing
Source: Chem Sci. 2021 Apr 5;12(19):6518–25. doi: 10.1039/d1sc01175h (PMC8132935; doi:10.1039/d1sc01175h)
Supplement: SC-012-D1SC01175H-s001 [file SC-012-D1SC01175H-s001.pdf]

Electronic Supplementary Material (ESI) for Chemical Science.
This journal is © The Royal Society of Chemistry 2021

Electronic Supplementary Information

Efficient Organic Host-Guest Room-Temperature Phosphorescence: Tunable Triplet-Singlet Crossing and Theoretical Calculation for Molecular Packing

Yunxiang Lei,‡[b] Junfang Yang,‡[c] Wenbo Dai,[a] Yisha Lan,[a] Jianhui Yang,[a] Xiaoyan Zheng,*[c] Jianbing Shi,[a] Bin Tong,[a] Zhengxu Cai,*[a] Yuping Dong.[a]

[a] School of Materials Science & Engineering, Beijing Institute of Technology, Beijing 100081, P. R. China.

[b] School of Chemistry and Materials Engineering, Wenzhou University, Wenzhou 325035, P. R. China.

[c] School of Chemistry and Chemical Engineering, Beijing Institute of Technology, Beijing 100081, P. R. China.

‡: These authors contribute equally.

1. Materials and characterization.

Both the host and guest molecules were purified by column chromatography twice, followed by recrystallization. ^{1}H and ^{13}C NMR spectra were carried out by Bruker ARX400 spectrometer with $CDCl_3$ as the solvent. Mass spectra were performed by using Finnigan BIFLEX III mass spectroscopy. UV-vis absorption spectra were measured by Persee TU-1901. Fluorescence spectra were measured by using Hitachi F-7000 spectrophotometer. Phosphorescence spectra were measured by using FLS920 lifetime and steady state spectrometer. XRD was recorded using an Empyrean X-ray diffraction instrument. X-ray crystal structure analyses were measured by using Bruker-AXS SMART APEX2 CCD diffractometer. Solid-state emission quantum yields (Φ) were collected by using FluoroMax-4 (Horiba Jobin Yvon) fluorimeter equipped with integrated sphere. The fluorescence decay data were analyzed using DAS-6 Fluorescence Decay Analysis software with 2 exponential analysis program. The quality of the exponential fits was evaluated by the $\chi 2$ of <2.0.

2. General procedure for the synthesis of target compounds.

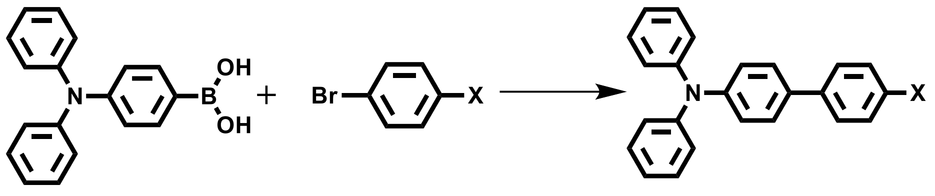

X = H, CH_3, OCH_3, $N(CH_3)_2$

Scheme S1. Synthetic route of **DBA, MDBA, MODBA**, and **MADBA**

Synthesis and characterization: DBA, MDBA, MODBA, and **MADBA** were synthesized by Suzuki reaction. To the solution of (4-(diphenylamino)phenyl)boronic acid (10.0 mmol), bromobenzene derivatives (12.0 mmol), $Pd(PPh_3)_4$ (5.0 mol%), K_2CO_3 (5.0 mol%), in toluene (15.0 mL) and methanol (5.0 mL) solution bubbled with nitrogen. The mixture was stirred for 12 h at 80 °C under nitrogen atmosphere. Then the reaction solution was cooled to room temperature, $Pd(PPh_3)_4$ was filtered. After removal of solvent under reduced pressure, the crude products were purified by column chromatography (petroleum ether/ethyl acetate) to afford product.

DBA, white solid, 84.1% yield. ^1H NMR (400 MHz, $CDCl_3$): δ 7.55 - 7.57 (2H), 7.45 - 7.46 (2H), 7.38 - 7.42 (2H), 7.23 - 7.14 (11H), 7.00 - 7.04 (2H). ^{13}C NMR (101 MHz, $CDCl_3$): δ 147.74, 147.22, 140.70, 135.20, 129.31, 129.23, 128.76, 127.80, 126.84, 126.69, 124.46, 122.96, 123.96. ESI (m/z): calcd for $C_{24}H_{19}N$, 321.15. Found: 322.16.

MDBA, white solid, 86.3% yield. ^1H NMR (400 MHz, $CDCl_3$): δ 7.51 - 7.54 (4H), 7.18 - 7.34 (12H), 7.06 -7.10 (2H), 2.44 (3H). ^{13}C NMR (101 MHz, $CDCl_3$) δ 147.81, 146.96, 137.85, 136.57, 135.28, 129.51, 129.30, 127.63, 126.56, 124.38, 124.12, 122.87, 21.13. ESI (m/z): calcd for $C_{25}H_{21}N$, 335.17. Found: 336.16.

MODBA, white solid, 83.1% yield. ^1H NMR (400 MHz, $CDCl_3$): δ 7.55 - 7.54 (2H), 7.45 - 7.47 (2H), 7.15-7.31 (10H), 6.98 - 7.07 (4H), 3.87 (3H). ^{13}C NMR (101 MHz, $CDCl_3$) δ: 158.87, 147.80, 146.63, 135.05, 133.31, 129.26, 127.71, 127.36,

124.27, 122.78, 114.42, 55.35. ESI (m/z): calcd for $C_{25}H_{21}NO$, 351.16. Found: 352.17.

MADBA, white solid, 82.8% yield. ^{1}H NMR (400 MHz, $CDCl_3$): δ 7.48 - 7.54 (4H), 7.17 - 7.32 (10H), 7.04 - 7.07 (2H), 6.86 - 6.88 (2H), 3.04 (6H). ^{13}C NMR (101 MHz, $CDCl_3$) δ 149.57, 147.92, 146.05, 135.67, 129.24, 127.35, 126.97, 124.55, 124.13, 122.61, 113.08, 40.79. ESI (m/z): calcd for $C_{26}H_{24}N_2$, 364.19. Found: 365.27.

3. Photophysical properties.

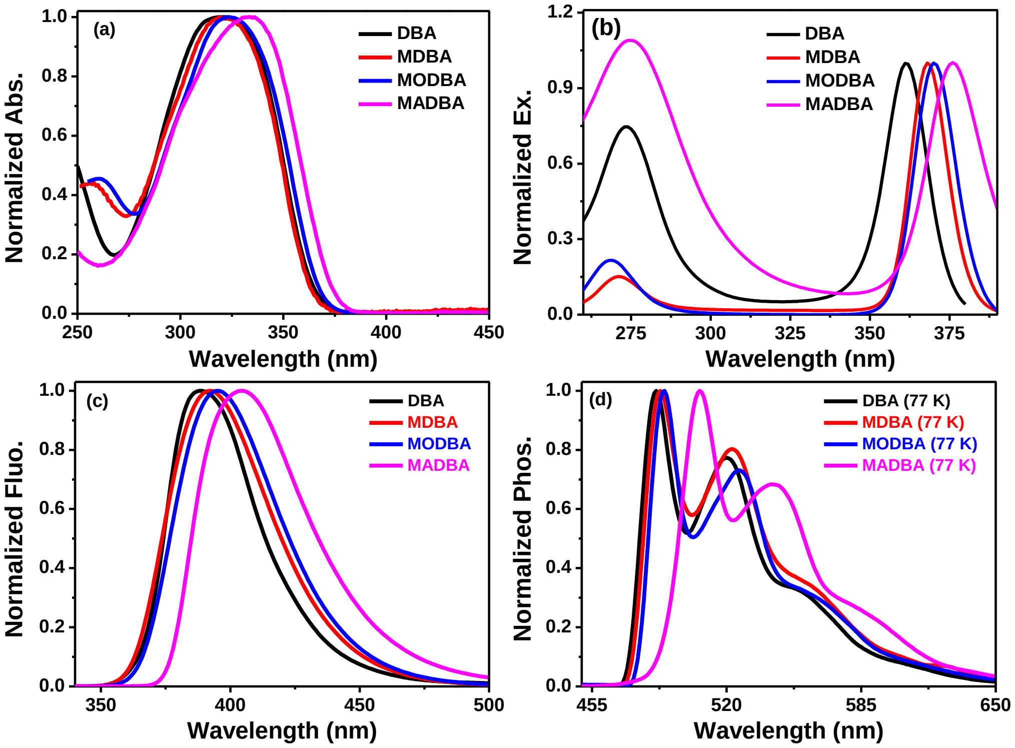

Figure S1. (a) Absorption spectra of guests in dichloromethane (Concentration: 1×10^{-5} mol/L). (b) Excitation spectra of guests in dichloromethane (Concentration: 1×10^{-5} mol/L). (c) Fluorescence spectra of guests in dichloromethane (Concentration: 1×10^{-5} mol/L). Excitation wavelength: 330 nm (d) Phosphorescence spectra of guests in dichloromethane at 77 K (Concentration: 1×10^{-4} mol/L). Excitation wavelength: 360 nm.

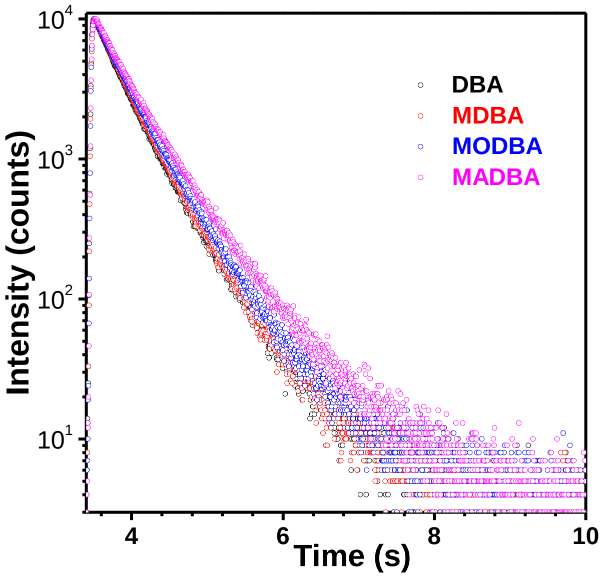

Figure S2. Phosphorescence decay curves of guests in dichloromethane at 77 K (Concentration: 1×10^{-4} mol/L). Excitation wavelength: 360 nm, Delayed time: 5 ms.

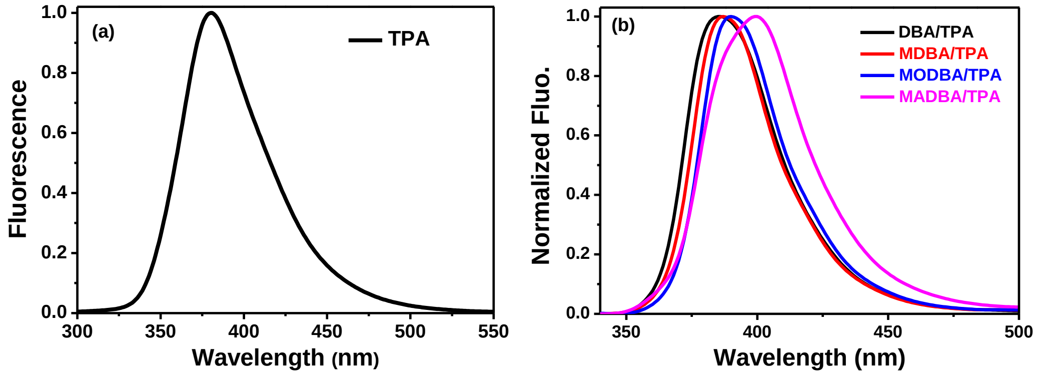

Figure S3. (a) Fluorescence spectra of **TPA** in crystalline state. Excitation wavelength: 280 nm. (b) Fluorescence spectra of doping powders. Excitation wavelength: 320 nm.

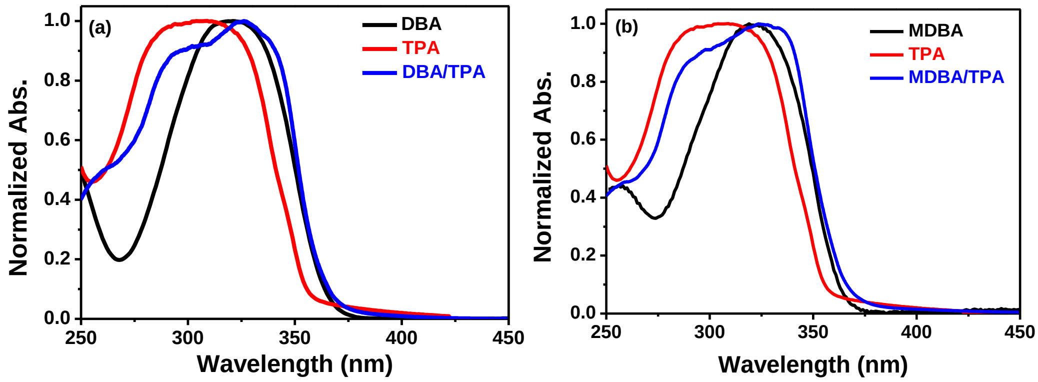

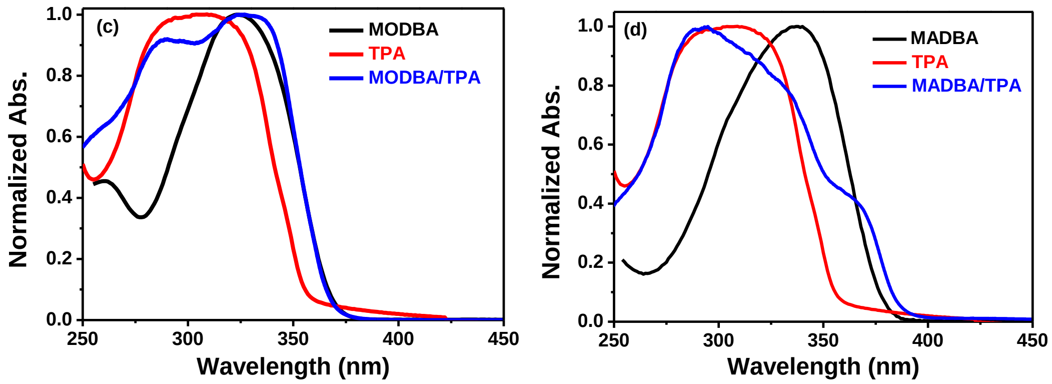

Figure S4. Absorption spectra of guest powders, host powder, and their doping powders.

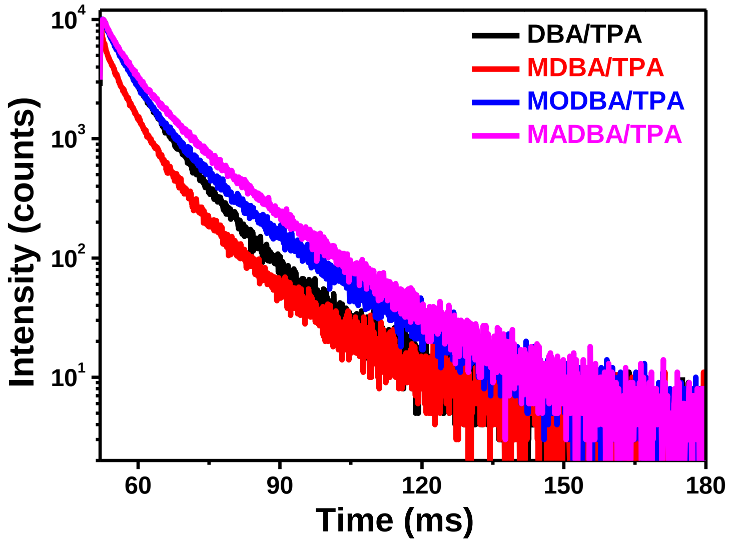

Figure S5. TADF decay curves of the four doping materials at 400 nm

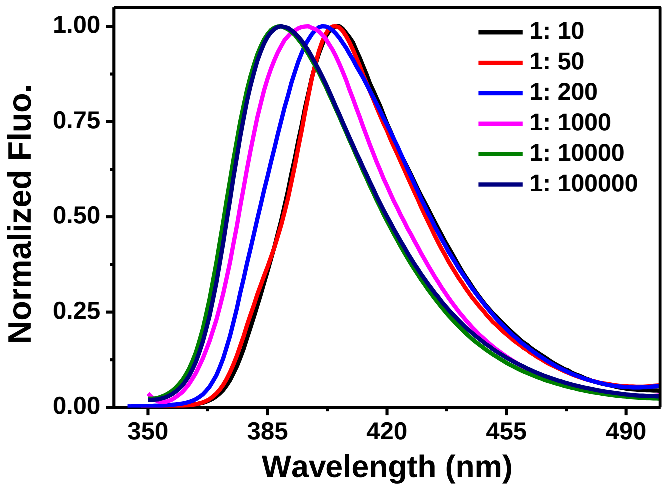

Figure S6. Fluorescence spectra of the **MADBA/TPA** powders with different amount of **MADBA** (Molar ratio).

4. Molecular dynamics (MD) simulations.

The atom types and parameters of **MADBA** and **TPA** were built from the general AMBER force field. To obtain the partial charge of each atom of two molecules, the electrostatic potential of two molecules were calculated at the B3LYP/6-31G* level based on the optimized geometric structures using Gaussian 16 package[1]. The partial charges of the atoms reproducing the electrostatic potential of two molecules were obtained by using restrained electrostatic potential (RESP)[2, 3] fit method. In order to simulate **MADBA/TPA** system, we firstly built a 4.6×3.1×4.4 nm **TPA** unit cell with 192 **TPA** molecules, and then a **MADBA** molecule was placed in the position vacated by the two host molecules to obtain the **MADBA/TPA** model as an initial model. For MD simulations, firstly, the energy minimization were performed by using steepest descent algorithm and the conjugate gradient, then we performed the 500 ps MD simulations under the NVT (P = 1 bar, T = 10 K) ensemble. The temperature was controlled by the velocity rescaling thermostat[4]. Then we performed the 10 ns MD simulations under the NPT (P = 1 bar, T = 10 K) ensemble coupled by Parrinello-Rahman barostat scheme[5]. The Newton's classical equations of motion were integrated at a time step of 2 fs using the classical leapfrog algorithm. Trajectory analysis was done with the help of utility tools included in the GROMACS (version 5.1.5)[6] and VMD[7] packages.

5. QM/MM calculations.

Both doping system and **MADBA** crystal were further studied by QM/MM calculations to consider the influence of different external environments on the luminescence properties of **MADBA**. Herein, QM/MM calculations were performed by ONIOM model[8] in Gaussian 16 package[1]. All geometric structures of ground (S_0) and excited (S_1/T) states were obtained by DFT and TD-DFT, respectively. In addition, frequencies for all optimized structures were evaluated to check the absence of imaginary frequencies. The ONIOM models[8] for **MADBA/TPA** doping system and **MADBA** crystal were both partitioned into two layers. **MADBA** molecule in the **MADBA/TPA** system and in the center of **MADBA** crystal was selected as

high-layer and treated by B3LYP functional with the 6-31G** basis set, and other molecules were set as low-layer and treated by universal force field[9]. Atoms in the high-layer are fully relaxed for two systems, while others are frozen. The QM region provides key information of the electronic excited states, while the MM region includes environment effect. The electrostatic embedding scheme with QM polarization was adopted.[10]

6. Spin-orbit couplings (SOCs) calculations.

In addition, the SOCs constants were calculated between S_1 and low-lying triplet states at the B3LYP/6-31G** level by using ORCA program[11] to prove the easier ISC process of the doping system compared to pure MADBA crystal.

1. M. J. Frisch GWT, H. B. Schlegel, G. E. Scuseria, M. A. Robb, J. R. Cheeseman, G. Scalmani, V. Barone, G. A. Petersson, H. Nakatsuji, X. Li, M. Caricato, A. V. Marenich, J. Bloino, B. G. Janesko, R. Gomperts, B. Mennucci, H. P. Hratchian, J. V. Ortiz, A. F. Izmaylov, J. L. Sonnenberg, D. Williams-Young, F. Ding, F. Lipparini, F. Egidi, J. Goings, B. Peng, A. Petrone, T. Henderson, D. Ranasinghe, V. G. Zakrzewski, J. Gao, N. Rega, G. Zheng, W. Liang, M. Hada, M. Ehara, K. Toyota, R. Fukuda, J. Hasegawa, M. Ishida, T. Nakajima, Y. Honda, O. Kitao, H. Nakai, T. Vreven, K. Throssell, J. A. Montgomery, Jr., J. E. Peralta, F. Ogliaro, M. J. Bearpark, J. J. Heyd, E. N. Brothers, K. N. Kudin, V. N. Staroverov, T. A. Keith, R. Kobayashi, J. Normand, K. Raghavachari, A. P. Rendell, J. C. Burant, S. S. Iyengar, J. Tomasi, M. Cossi, J. M. Millam, M. Klene, C. Adamo, R. Cammi, J. W. Ochterski, R. L. Martin, K. Morokuma, O. Farkas, J. B. Foresman, and D. J. Fox. Gaussian16 Revision A.03. Gaussian Inc. Wallingford CT. (2016.).
2. Bayly CI, Cieplak P, Cornell W, Kollman PA. A well-behaved electrostatic potential based method using charge restraints for deriving atomic charges: the RESP model. *J Phys Chem* **97**, 10269-10280 (1993).

3. Cornell WD, Cieplak P, Bayly CI, Kollmann PA. Application of RESP Charges to Calculate Conformational Energies, Hydrogen Bond Energies, and Free Energies of Solvation. *J Am Chem Soc* **115**, 9620-9631 (1993).
4. Bussi G, Donadio D, Parrinello M. Canonical sampling through velocity rescaling. *J Chem Phys* **126**, 014101 (2007).
5. Parrinello M, Rahman A. Polymorphic Transitions in Single-Crystals-a-New Molecular-Dynamics Method. *J Appl Phys* **52**, 7182-7190 (1981).
6. Abraham MJ, Spoel Dvd, Lindahl E, Hess B. GROMACS User Manual version 5.1. (2015).
7. Humphrey W, Dalke A, Schulten K. VMD: Visual molecular dynamics. *J Mol Graph* **14**, 33-38 (1996).
8. Dapprich S, Komáromi I, Byun KS, Morokuma K, Frisch MJ. A new ONIOM implementation in Gaussian98. Part I. The calculation of energies, gradients, vibrational frequencies and electric field derivatives1Dedicated to Professor Keiji Morokuma in celebration of his 65th birthday.1. *J Mol Struct : THEOCHEM* **461-462**, 1-21 (1999).
9. Rappe AK, Casewit CJ, Colwell KS, Goddard WA, Skiff WM. UFF, a full periodic table force field for molecular mechanics and molecular dynamics simulations. *J Am Chem Soc* **114**, 10024-10035 (1992).
10. Bakowies D, Thiel W. Hybrid Models for Combined Quantum Mechanical and Molecular Mechanical Approaches. *J Phys Chem* **100**, 10580-10594 (1996).
11. Neese, F. Wennmohs, F. Becker, U. Riplinger, C. The ORCA quantum chemistry program package. *J Chem Phys* **152**, 224108-224126 (2020).

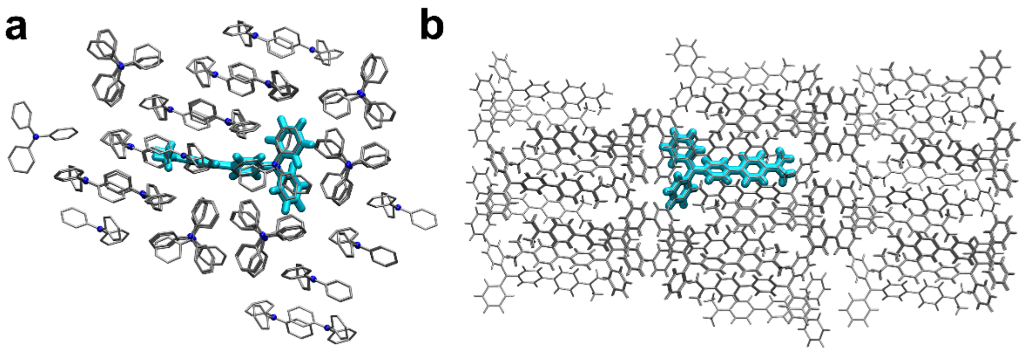

Figure S7. The representative of QM/MM model for the doping system (left) and MADBA crystalline state (right).

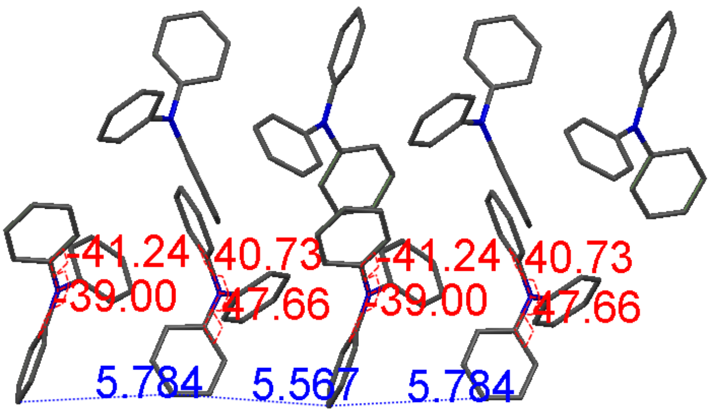

Figure S8. Molecular packing along the b-axis in **TPA** single crystal

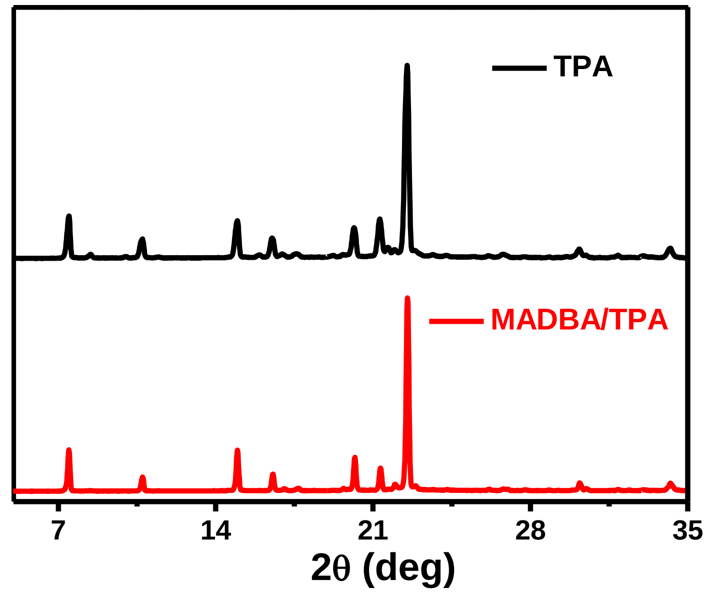

Figure S9. The XRD curves spectra of **TPA** sample and **MADBA/TPA** doping material.

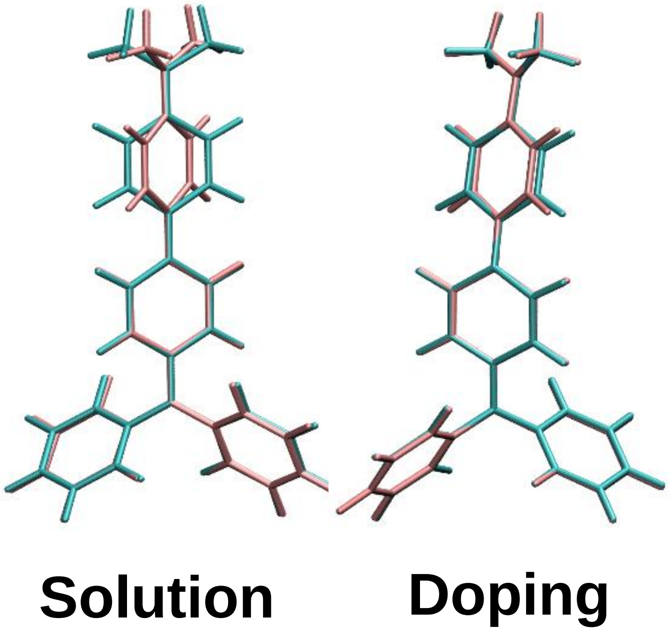

Figure S10. Superposition of optimized structures at both S_0 and T_1 states for **MADBA** in solution state and doping state.

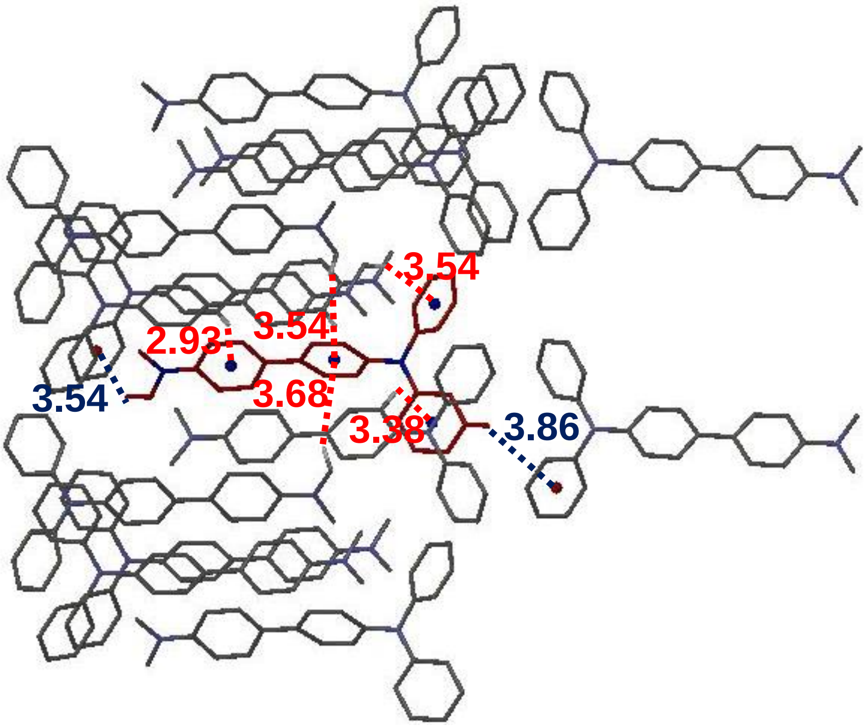

Figure S11. The interaction distance of C-H···π between **MADBA** molecules in crystal.

Table S1. Selected bond lengths (Å), bond angle (degree), and dihedral angle (degree) of **MADBA** at S_0 (S_1, T_1) minimum for **MADBA** in solution.

	S_0	S_1	T_1	$\lvert\Delta(S_0-S_1)\rvert$	$\lvert\Delta(S_0-T_1)\rvert$
C5-C20	1.48	1.43	1.41	0.05	0.07
C4-C5-C20	121.46	122.45	122.69	0.98	1.22
C5-C20-C21	121.75	122.77	122.95	1.02	1.21
C6-C5-C20	121.53	122.45	122.68	0.92	1.15
C5-C20-C25	121.79	122.77	122.94	0.98	1.15
C7-C6-C5-C20	-179.38	-179.88	-179.76	0.50	0.37
C4-C5-C20-C21	33.30	4.63	1.84	28.67	31.47
C4-C5-C20-C25	-147.02	-175.38	-178.00	28.36	30.99
C6-C5-C20-C25	32.89	4.62	1.99	28.27	30.91
C6-C5-C20-C21	-146.70	-175.37	-178.17	28.67	31.48
C5-C20-C21-C22	179.91	179.95	179.53	0.04	0.38
C5-C20-C25-C24	-179.91	-179.95	-179.65	0.04	0.26

Table S2. Selected Bond Lengths (Å), Bond Angle (degree), and Dihedral Angle (degree) of MADBA at S_0 (S_1, T_1) minimum for **MADBA** in doping.

	S_0	S_1	T_1	$\|\Delta(S_0-S_1)\|$	$\|\Delta(S_0-T_1)\|$
C5-C20	1.48	1.44	1.40	0.04	0.08
C4-C5-C20	120.86	121.29	121.87	0.43	1.01
C5-C20-C21	120.51	121.71	122.22	1.20	1.71
C6-C5-C20	121.99	122.61	122.93	0.62	0.94
C5-C20-C25	123.09	123.18	123.23	0.08	0.14
C7-C6-C5-C20	-168.59	-165.69	-161.93	2.90	6.65
C4-C5-C20-C21	-26.51	-13.57	-9.60	12.94	16.90
C4-C5-C20-C25	156.25	168.48	171.88	12.23	15.63
C6-C5-C20-C25	-29.94	-17.36	-14.25	12.58	15.69
C6-C5-C20-C21	147.30	160.59	164.27	13.29	16.96
C5-C20-C21-C22	-176.51	-176.71	-177.27	0.20	0.76
C5-C20-C25-C24	175.99	177.25	178.37	1.26	2.38

6. NMR Spectra

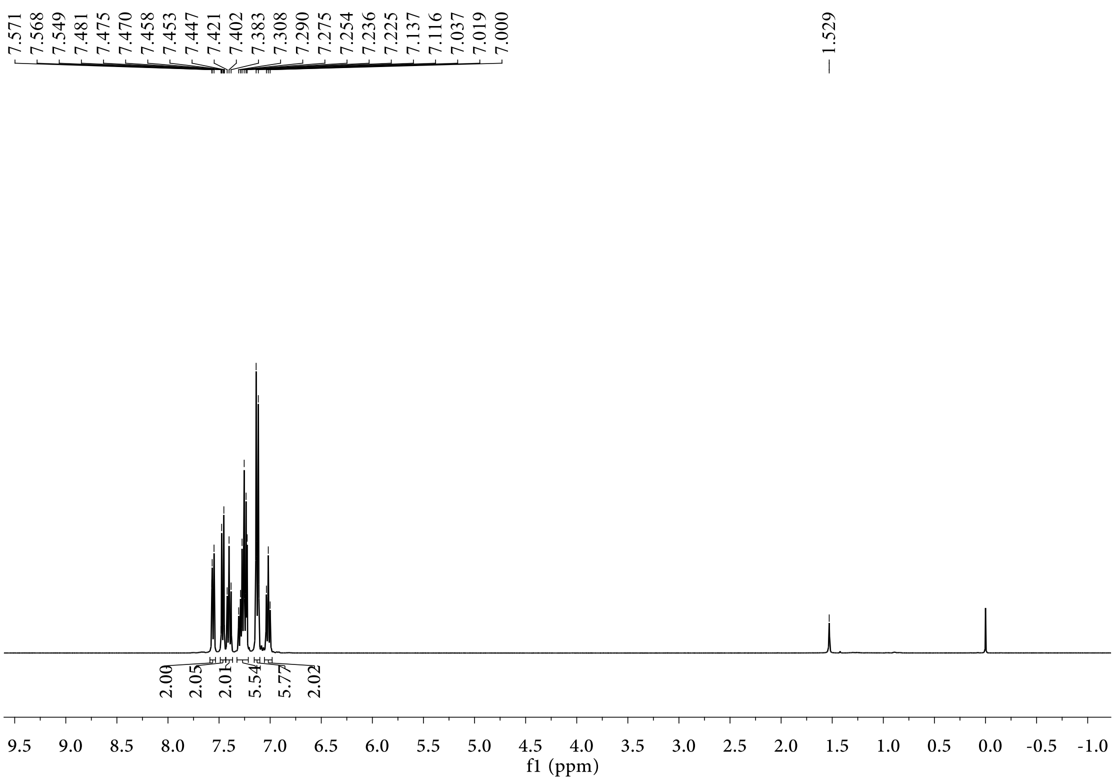

Figure S12. The ^{1}H NMR spectra of **DBA** in $CDCl_3$, 298 K.

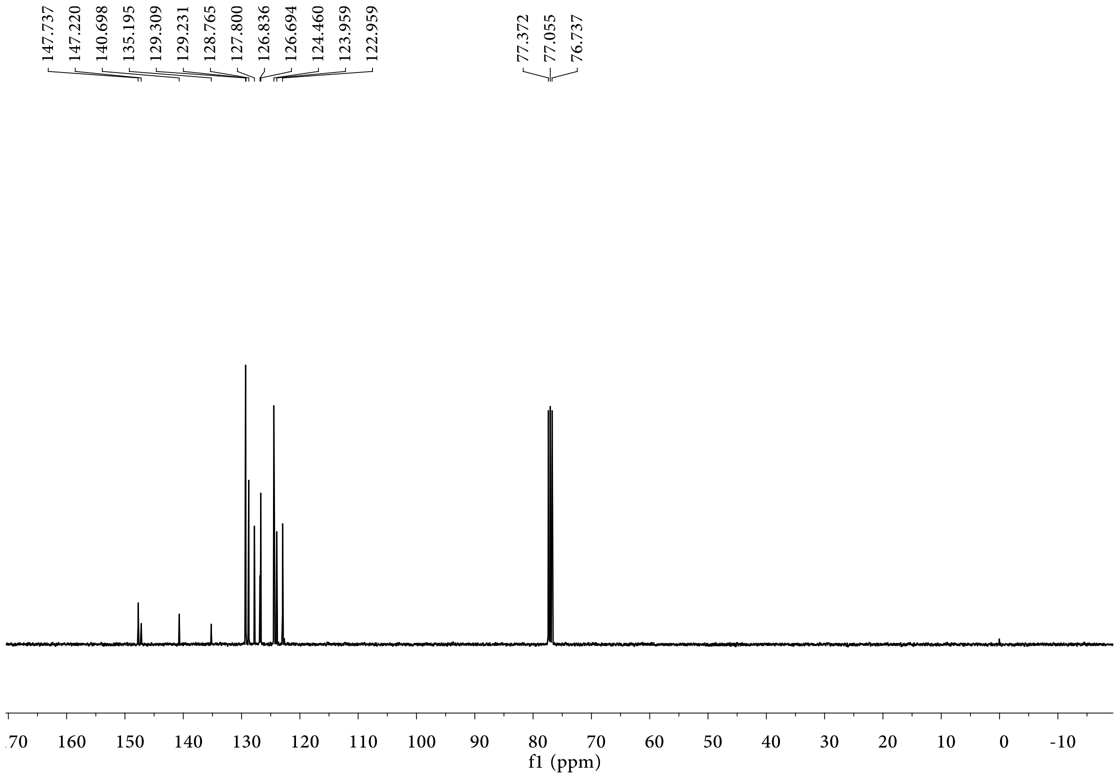

Figure S13. The ^{13}C NMR spectra of **DBA** in $CDCl_3$, 298 K.

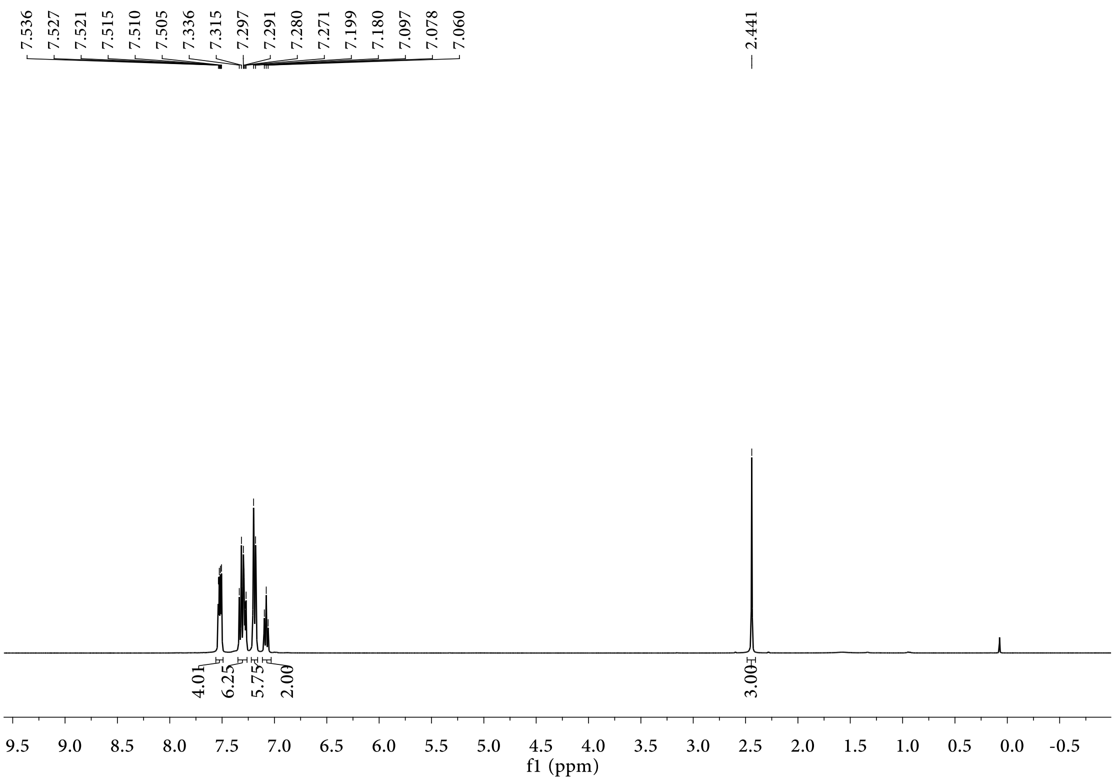

Figure S14. The ^{1}H NMR spectra of **MDBA** in $CDCl_3$, 298 K.

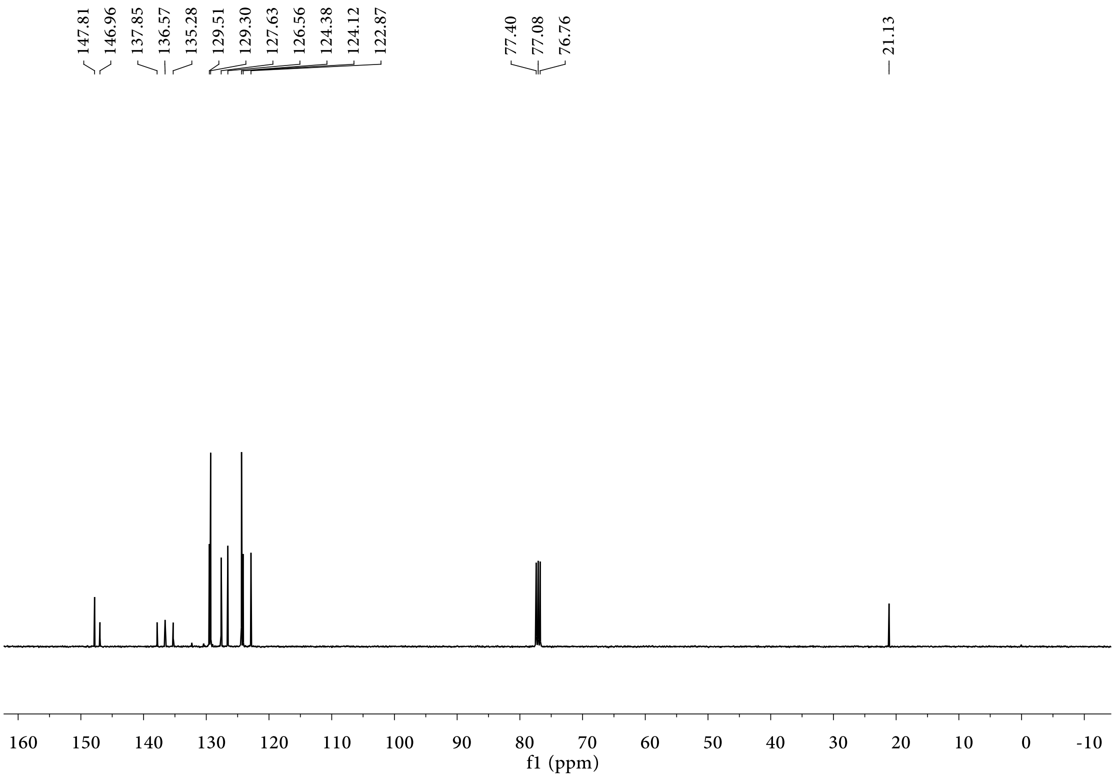

Figure S15. The ^{13}C NMR spectra of **MDBA** in $CDCl_3$, 298 K.

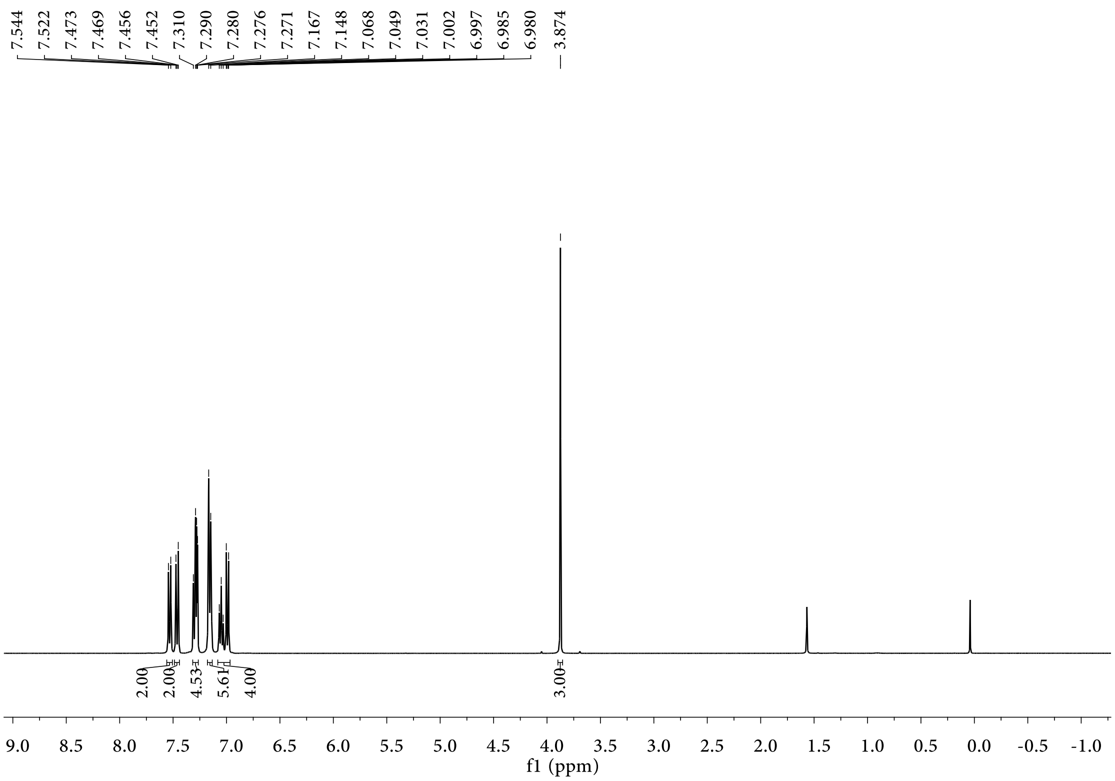

Figure S16. The ^{1}H NMR spectra of **MODBA** in $CDCl_3$, 298 K.

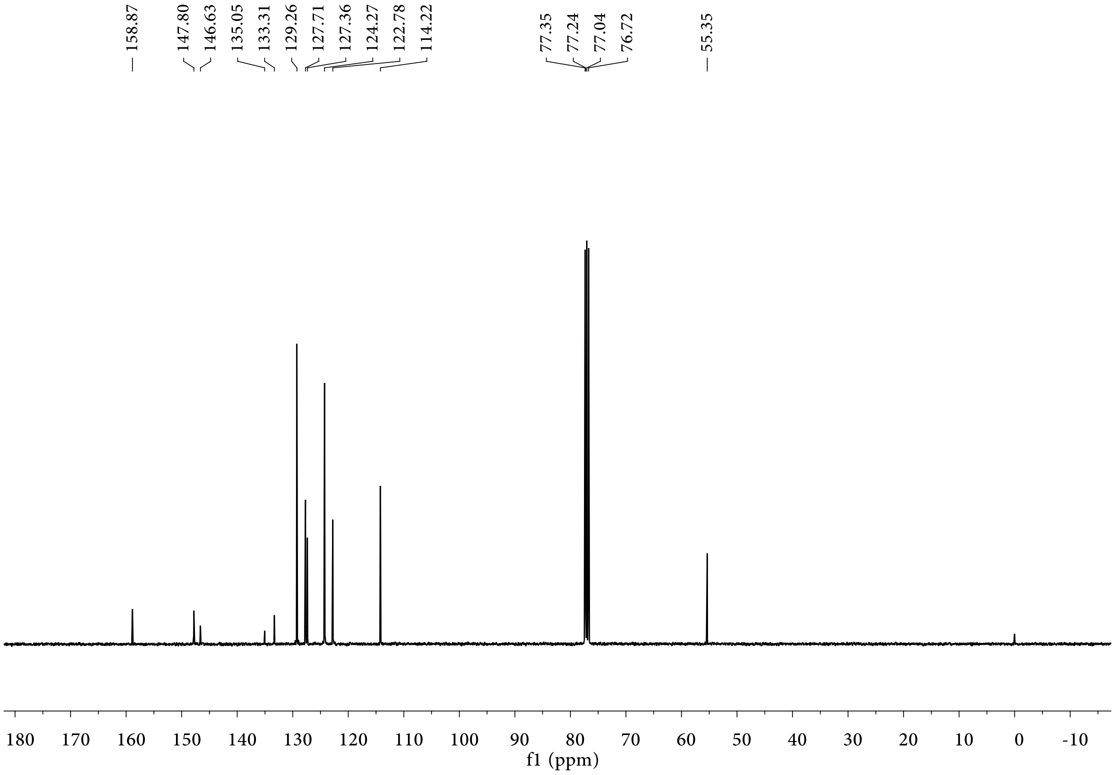

Figure S17. The ^{13}C NMR spectra of **MODBA** in $CDCl_3$, 298 K.

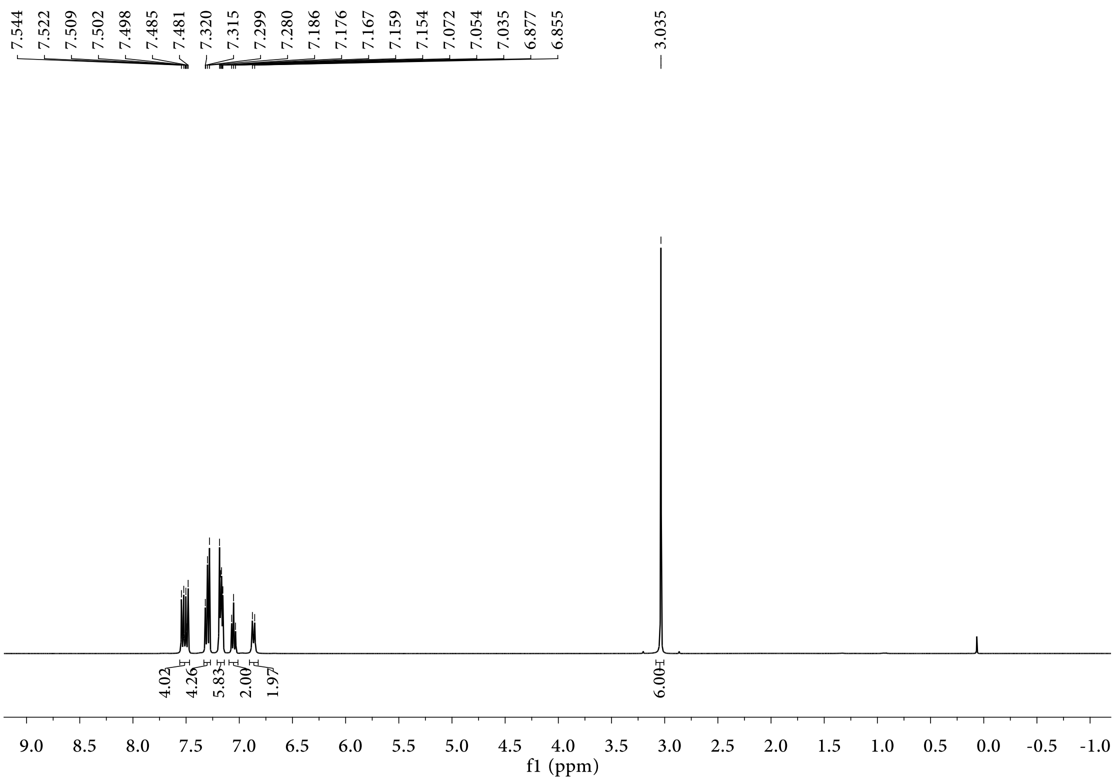

Figure S18. The ^{1}H NMR spectra of **MADBA** in $CDCl_3$, 298 K.

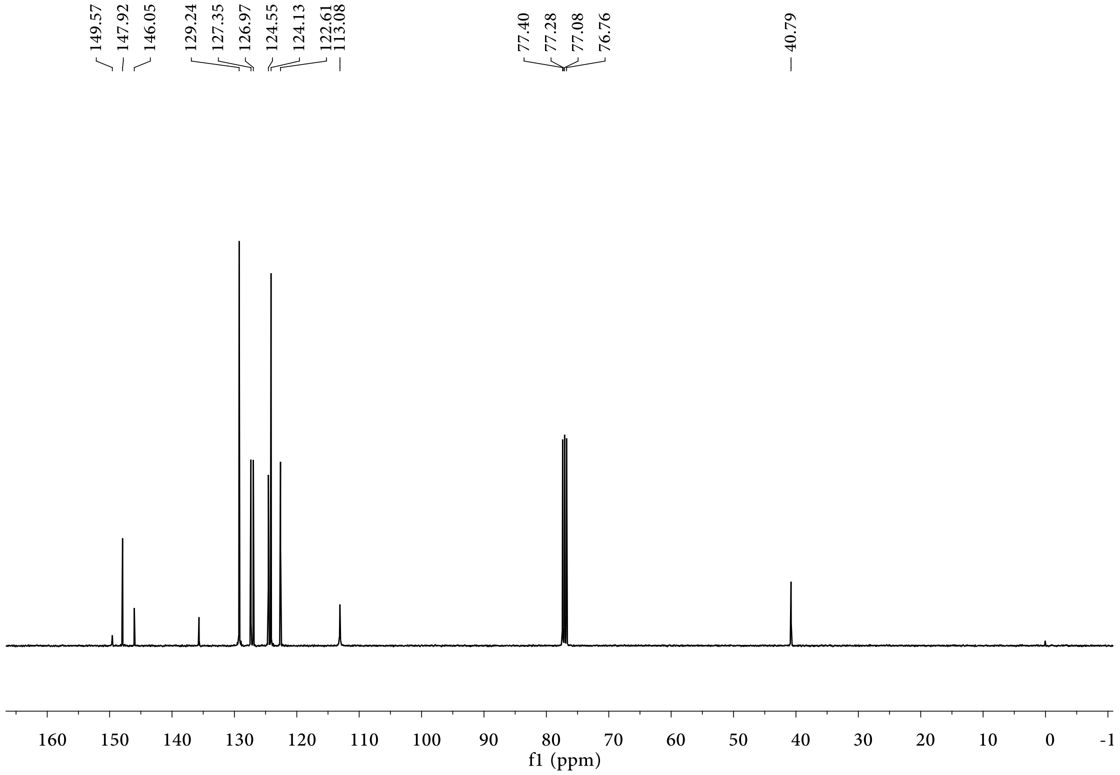

Figure S19. The ^{13}C NMR spectra of **MADBA** in $CDCl_3$, 298 K.
